# Polar Polymorphism: A New Intermediate Structure toward the Thin-Film Phase in Asymmetric Benzothieno[3,2-*b*][1]-benzothiophene Derivatives

**DOI:** 10.1021/acs.chemmater.3c02926

**Published:** 2023-12-26

**Authors:** Shunya Yan, David Cornil, Jérôme Cornil, David Beljonne, Rogger Palacios-Rivera, Carmen Ocal, Esther Barrena

**Affiliations:** † Instituto de Ciencia de Materiales de Barcelona (ICMAB-CSIC), Campus UAB, Bellaterra, E-08193 Barcelona, Spain; ‡ Laboratory for Chemistry of Novel Materials, University of Mons (UMONS), 20 Place du Parc, 7000 Mons, Belgium

## Abstract

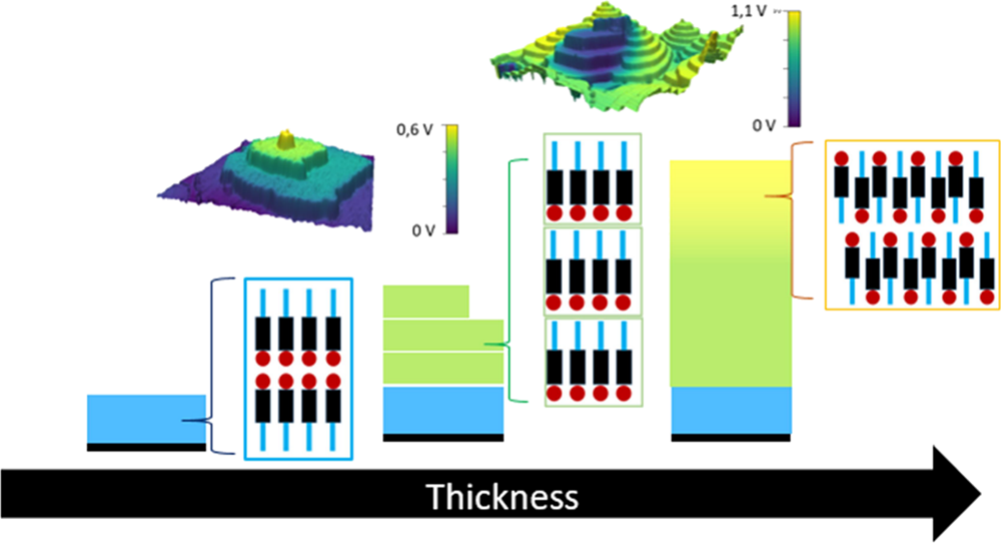

Understanding structure and polymorphism is relevant for any organic device optimization, and it is of particular relevance in 7-decyl-2-phenyl[1]benzothieno[3,2-*b*][1]benzothiophene (Ph-BTBT-10) since high carrier mobility in Ph-BTBT-10 thin films has been linked to the structural transformation from the metastable thin-film phase to the thermodynamically stable bilayer structure via thermal annealing. We combine here a systematic nanoscale morphological analysis with local Kelvin probe force microcopy (KPFM) that demonstrates the formation of a polar polymorph in thin films as an intermediate structure for thicknesses lower than 20 nm. The polar structure develops with thickness a variable amount of structural defects in the form of individual flipped molecules (point defects) or sizable polar domains, and evolves toward the reported nonpolar thin-film phase. The direct experimental evidence is supported by electronic structure density functional theory calculations. The structure of the film has dramatic effects on the electronic properties, leading to a decrease in the film work function (by up to 1 eV) and a considerable broadening of the occupied molecular orbitals, attributed to electrostatic disorder. From an advanced characterization point of view, KPFM stands out as a valuable tool for evaluating electrostatic disorder and the conceivable emergence of polar polymorphs in organic thin films. The emergence of polar assemblies introduces a critical consideration for other asymmetric BTBT derivatives, which may be pivotal to understanding the structure–property relationships in organic field-effect transistors (OFETs). A precise determination of any polar assemblies close to the dielectric interface is critical for the judicious design and upgrading of high-performance OFETs.

## Introduction

In recent years, significant progress has been made in the development of novel π-conjugated organic materials used as active layer in high-performance organic electronic devices.^1−4^ Heteroarenes with fused aromatic rings, such as [1]benzothieno[3,2-*b*]benzothiophene (BTBT) derivatives, qualify among the best-performing organic semiconductors for field-effect transistors (OFETs) with a charge carrier mobility over 10 cm^2^ s^–1^ V^–1^ and excellent air stability.^5−7^ Further applications extend to photodetectors/phototransistors or to their use as donors to form organic charge-transfer (CT) complexes.^5,6^ Furthermore, the high designability of BTBT derivatives has enabled the formation of a variety of symmetric and/or asymmetric alkylated BTBT molecular structures.^8−13^

Besides their role in enhancing molecular solubility, long alkyl chain side groups serve to improve the intermolecular packing, leading to a lamella-like structure (where the interlayer distance correlates with the length of the molecule) and a herringbone (HB) arrangement of the π-cores that allows for efficient π–π overlap between adjacent molecules. Indeed, the excellent charge transport properties of OFETs based on thin films of dialkyl BTBT derivatives can be attributed to the layered crystalline structure consisting of an alternating stack of alkyl chain and BTBT core layers.^5^ Furthermore, the synthesis of asymmetric BTBT derivatives with nonidentical side groups, has prompted a large number of studies because they offer greatly improved thermal stability and liquid crystalline (LC) phases that can be used to engineer the morphology of the films.^10,14−17,19^ While van der Waals forces are the dominant interactions between nonpolar molecules, dipolar electrostatic interactions also come into play for building blocks with an unbalanced distribution of the electronic density.

**Figure 1:**
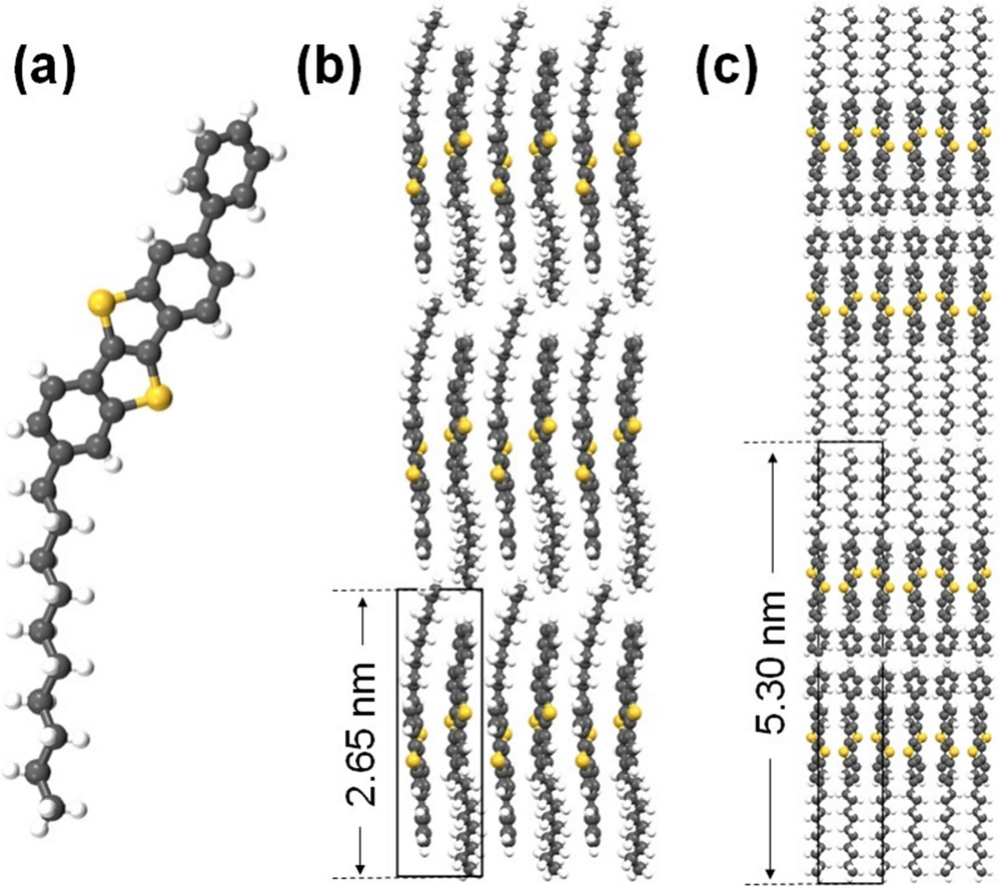
(a) Molecular structure of Ph-BTBT-10. (b) Thin-film polymorph consisting of a stack of single layers with antiparallel orientation of the molecules.^25^ (c) Bilayer (BL) lamellar packing consisting of a stack of double layers, where the molecules have a unidirectional orientation within each layer but are arranged antiparallel to the molecules in the adjacent layer.^25^ The corresponding stacking periodicities are indicated.

In particular, the monoalkylated 7-decyl-2-phenyl[1]benzothieno [3,2-*b*][1]benzothiophene (Ph-BTBT-10) is a fascinating BTBT derivative that has gained significant attention due to its excellent high carrier mobility in p-type OFETs.^10,14,18^ LC phases emerging at higher temperatures (*T* > 145 °C)^23−25^ can be exploited for morphological and structural control. A key to the high performance of Ph-BTBT-10 in OFETs is the bilayer arrangement. As determined by single-crystal X-ray diffraction (XRD),^26^ in the bilayer structure the Ph-BTBT-10 molecules stack on top of each other in a head-to-head (or tail-to-tail) fashion, resulting in a stacking periodicity of *d*_BL_ = 5.30 nm, i.e., twice the molecular length (Figure 1).^23−26^ The molecules are unidirectionally oriented within each half-bilayer, with the BTBT cores adopting an in-plane HB organization similar to that for symmetric derivatives. As a result of the asymmetric nature of the Ph-BTBT-10 and the associated electrical dipole, each half-bilayer is polar, whereas the entire BL is nonpolar. The HB packing of the molecular π-cores favors the two-dimensional transport and affords high carrier mobility.^14,26,27^

During thin-film growth, the development of metastable crystalline structures that differ from the bulk structures is a widespread phenomenon in organic materials,^28^ that also takes place in Ph-BTBT-10. Unlike single crystals, for films grown at room temperature (RT), the molecules have been found to adopt a layered structure with an interlayer spacing of *d*_TF_ = 2.65 nm, i.e., corresponding to a packing of single molecular layers (SL) and referred to as a thin-film phase. The high carrier mobility in OFETs based on Ph-BTBT-10 thin films has been linked to the structural transformation from the metastable thin-film phase to the thermodynamically stable BL stacking via thermal annealing at *T* > 110 °C, rendering approximately ten times larger mobility.^14^ The formation of SL and BL structures as well as the structural transition between them by thermal annealing has also been found for other monoalkylated BTBT derivatives^11,15,29−34^ and other asymmetric materials,^35,36^ suggesting that they are common among asymmetric molecules.

The molecular packing in the thin-film structure was under debate until the structure was finally determined by wide-angle X-ray scattering in a study carried out on 60 nm-thick films.^21^ The reported structure is presented in Figure 1b. Molecules in the thin-film polymorph maintain a HB arrangement of the conjugated units and are packed with a slightly offset antiparallel orientation within each layer. The antiparallel orientation of the molecules in the plane yields a nonpolar layer as does the entire bilayer. As can be inferred from the structural models, the alignment of the BTBT cores in the BL (Figure 1c) favors a better overlap of the π-molecular orbitals than for the shifted antiparallel arrangement of the molecules in the thin-film phase (Figure 1b).

Given that the carrier mobility of the films is crucially dependent on the formed polymorph, many studies have focused on establishing strategies to control it via growth and thermal treatments, including recrystallization from the smectic phase, and in gaining a deep understanding of the molecular processes underlying the temperature-induced structural transformations.^10,14,22,37−41^ Still a complete perception of the effect of the substrate interface on the origin and variation of polymorphic phases has not yet been obtained. It remains unexplored how the electronic properties of thin films are affected by structural changes.

We combine here a systematic nanoscale morphological analysis with local Kelvin probe force microcopy (KPFM) that demonstrates the formation of single layers during the first stages of the growth (for thicknesses lower than 20 nm), which develop as an intermediate structure toward the thin-film phase as thickness increases. The experimental evidence is supported by electronic structure density functional theory (DFT) calculations, highlighting the effects of polar polymorphism on the electronic properties of the films. The thickness dependence of the reported polar structure and the potential coexistence of diverse polymorphs represent other key factors to be considered for controlling the efficiency and functionality of organic devices.

## Results

### Low Coverage: Demonstration of Polar Assemblies

Throughout this work, we establish the correlation between organic film topography and surface potential (SP) obtained by KPFM. The SP map provides a measure of spatial variation of the sample work function. For the setup employed here (see Experiments and Methods section), regions with a more positive value of SP correspond to a lower value of the work function; in other words, to a downshift of the vacuum level (*E*_vac_). We report here common characteristics derived from a systematic investigation performed for Ph-BTBT-10 films (Figure 1a) deposited from the vapor phase onto the native oxide surface of Si(100) substrates, under high vacuum conditions (10^–7^ mbar) and at different substrate temperatures below the transition from thin film to BL structure, ranging from 25 to 90 °C. Although the conclusions are common to the entire series of samples studied, some of the selected images correspond to growths at 80 and 90 °C, since a higher substrate temperature favors the formation of larger terraces and, consequently, a better discrimination of the local SP (Figure S1).

**Figure 2:**
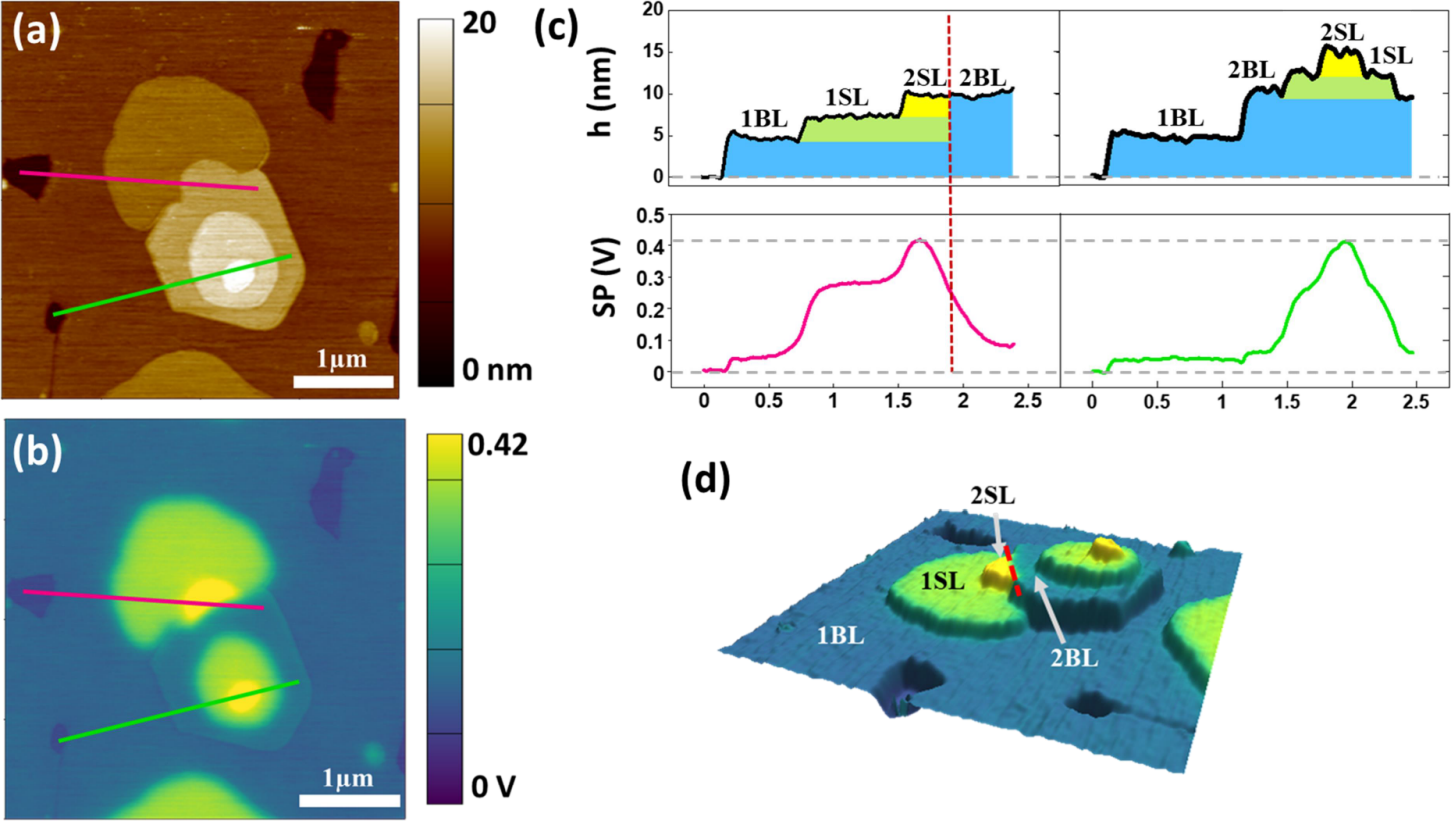
(a) Topographic image and (b) the corresponding surface potential map (raw data) for a nominal thickness of ≈5 nm of Ph-BTBT-10 deposited under UHV on the native oxide of a Si(100) substrate kept at 90 °C. (c) Line profiles obtained along the segments indicated in the corresponding topographic and SP images. For a clearer interpretation, the substrate level has been taken in the profiles as *h* = 0 and SP_Sub_ = 0. (d) Three-dimensional visualization of the merged topographic and SP data.

The first stages of the Ph-BTBT-10 growth are analyzed first. The amount of molecules deposited is given as the nominal thickness in nanometers (see Experiments and Methods section). Typical topographic and KPFM data obtained for a nominal thickness of ≈5 nm are shown in Figure 2. The observed surface morphology (Figure 2a), where only a few regions of the substrate (black) remain uncovered, confirms the formation of a nearly continuous molecular film with a thickness of ≈5 nm (dark brown). This observation is in agreement with the formation of a Ph-BTBT-10 bilayer in direct contact with the substrate,^21^ where molecules within each single layer are unidirectionally oriented but arranged antiparallel in one single layer with respect to the other. On top of the first bilayer (1BL), extremely flat stacked terraces indicate a crystalline layered growth. The line profile shown in the top right of Figure 2c, corresponding to the magenta segment in (a), indicates that on top of 1BL, there are two terraces with a step height of 2.65 nm ±0.05 nm. Given that, on average, the measured step height equals the molecular length of Ph-BTBT-10 (see Figure 1), each of such terraces is unambiguously ascribed to a single-molecule thick layer (1SL and 2SL). On the other hand, along the green profile in the top left of Figure 2c, one can see a terrace with bilayer thickness (marked as 2BL) followed by two more single layers. However, the most striking features are extracted from the SP maps. Outstandingly, BLs and SLs are clearly distinguished in the SP map (see Figure 2b and lower panels of Figure 2c). For simplicity, we assign the substrate as reference for SP (SP_Sub_ = 0 at the regions of the uncovered substrate). Note that BL regions exhibit the same value of SP, only differing by ≈0.05 V from SP_Sub_, i.e., stacked bilayers do not lead to potential buildup (see Figure 2b,c). Indeed, no surface dipole is expected for a stack of bilayers due to the nonpolar character of each BL and the absence of charge transfer between the substrate and the film (i.e., condition of vacuum level alignment). The same argument would apply for the stacking of nonpolar layers in the thin-film structure (Figure 1b). It is conversely observed in Figure 2c that the formation of 1SL leads to an increase of SP by ≈ + 0.25 V, indicating a net dipole in the SL configuration. As can be seen in the profiles at the bottom of Figure 2c, this quantitative effect is independent of the number of bilayers underneath, indicating that it is an intrinsic characteristic of the specific molecular arrangement within the SL. To facilitate visualization of the described effect, Figure 2d presents the 3D construction resulting from the superimposition of the SP data (color scale) to the topographic image. In summary, while SP remains constant regardless of the number of stacked BLs, SP becomes more positive and increases stepwise with the number of piled SLs. We note that this increase corresponds to a decrease in the local work function. Although from a morphological point of view, the stacking of an even number of SLs is indistinguishable from a stacking of BLs, the SP measurement provides a means of distinguishing the two types of crystalline structures. Very clarifying is the examination of the domain boundary between the two types of stacks signaled by a red dotted line in Figure 2c,d. While the domain boundary is clearly identified in the SP data, both domains are not distinguished in the topographic ones.

As mentioned above, the net electrical dipole observed for each SL is incongruent with the antiparallel orientation of molecules in the layer structure proposed for the thin-film phase determined for 60 nm-thick films.^25^ Conversely, the results obtained here for considerably thinner films are consistent with an assembly of unidirectionally oriented (polar) molecules in each SL. The positive accumulation of SP points unequivocally to the fact that the molecules of each of all stacked SLs are oriented with the alkyl chains facing up (see calculations below). In other words, the observed stacking consists of single molecular layers with the same molecular dipole orientation, such that their dipoles add up.

**Figure 3:**
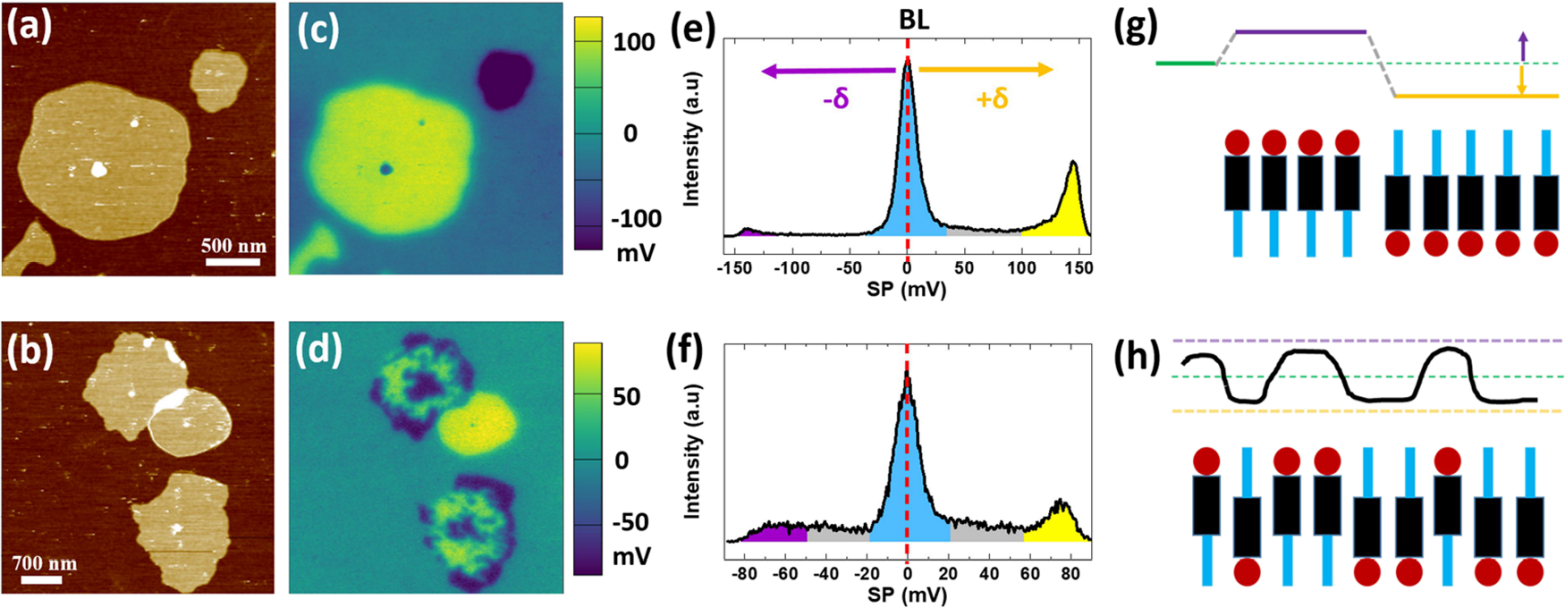
Topographic images (a,b) with the corresponding surface potential (SP) maps (c) and (d). Total *z* range in (a) is 8.5 nm (from the darkest to the brightest color). The histograms of each SP map are depicted in (e) and (f), respectively. In this case, in the absence of uncovered substrate, SP = 0 corresponds to the bilayer in contact with the substrate (1BL). The nominal thickness of Ph-BTBT-10 deposited is ≈6 nm. Illustrative diagrams of the vacuum level for single-molecule thick islands with unidirectional orientation (up or down) (g) and a mixed orientation (up and down) (h) of the molecules.

Although most of the observed SLs exhibit a positive SP, indicating that the chain-up configuration is the most frequent assembly, single layers with opposite electrical dipole orientations (chain-down) as well as layers with coexisting small domains (chain-up and chain-down) were also found. Figure 3 illustrates this observation for a nominal thickness of ≈6 nm. Topographic images (Figure 3a,b) were taken on two surface regions of the same sample that show several molecular islands with a rather rounded shape and identical height (≈ 2.7 nm) nucleated on top of the bilayer in contact with the substrate. We first analyze the SP map in Figure 3c, which shows that each individual island in Figure 3a exhibits a homogeneous SP value, but opposite sign to one another (yellow or dark blue on the color scale). That is, the molecular arrangement within each island causes an increase/decrease in the local SP by the same amount (δ = ± 0.15 eV) with respect to the underlying nonpolar BL (see the histogram in Figure 3e). Note that in this case, where no substrate is seen in the imaged area, the reference for SP has been taken at 1BL (blue peak in the histogram) while chain-down and chain-up islands lead to positive (yellow peak) and negative (purple peak) values, respectively. Each of the described islands is a single domain, indicating complete lateral phase separation of polarities in this case. The lower value of the SP change in this case (<0.2 V) may indicate some structural variability in the form of a certain amount of flipped molecules in the packing. Indeed, as illustrated in Figure 3b–d, lateral phase separation can occur in a much shorter range, giving rise to local electrostatic disorder. Next to the single domain circular island, two other islands do present an irregular shape and inhomogeneous SP. This observation reflects the coexistence of short-range assemblies down to the nanometer size with chain-down or chain-up molecules.

**Figure 4:**
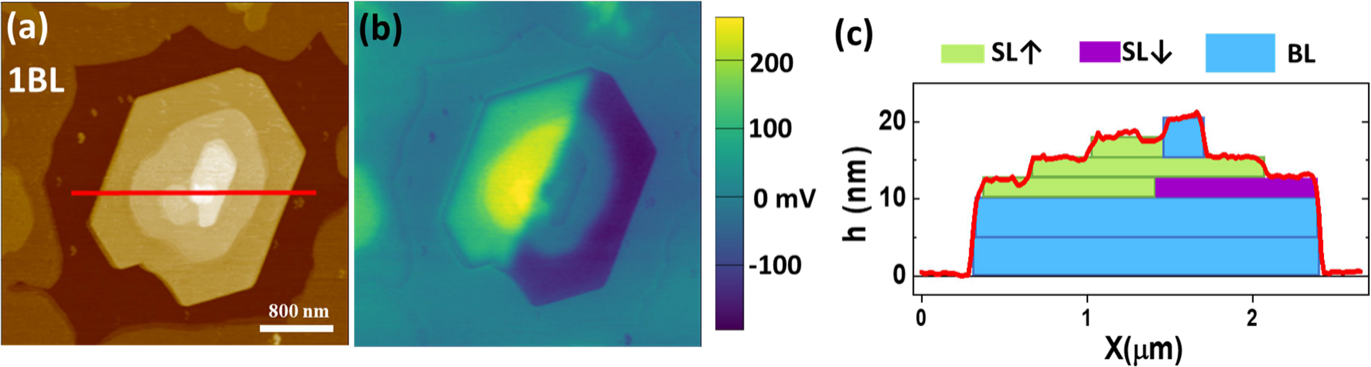
(a) Topographic image and (b) the corresponding surface potential (SP) for a nominal thickness of ≈5 nm of Ph-BTBT-10 deposited at 90 °C. (c) Line profile along the segment indicated in (a). The molecular packing of the different layers under the profile is represented by the respective colors, where SL↑ and SL ↓ indicate the direction of the alkyl chain.

The formation of laterally separated chain-up and chain-down domains gives rise to SP maps with features that appear uncorrelated with the surface morphology. We will return to this fact, but an attractive example of this scenario is shown in Figure 4. The electrostatic potential map of the crystallite at the center of the image is explained by the presence of a single molecular layer intercalated within the film with a domain boundary separating domains with chain-up and chain-down orientations. Subsequent polar layers (all chain-up molecules) grown on top of the stacking fault lead to such an asymmetric SP distribution. The proposed stacking scheme is provided with the line profile in Figure 4c. The selected example also illustrates that the nucleation of a BL at the mound top does not result in any modification of SP, i.e., the BL is nonpolar and does not contribute to SP.

### Evolution with Thickness: Molecular Flipping

**Figure 5:**
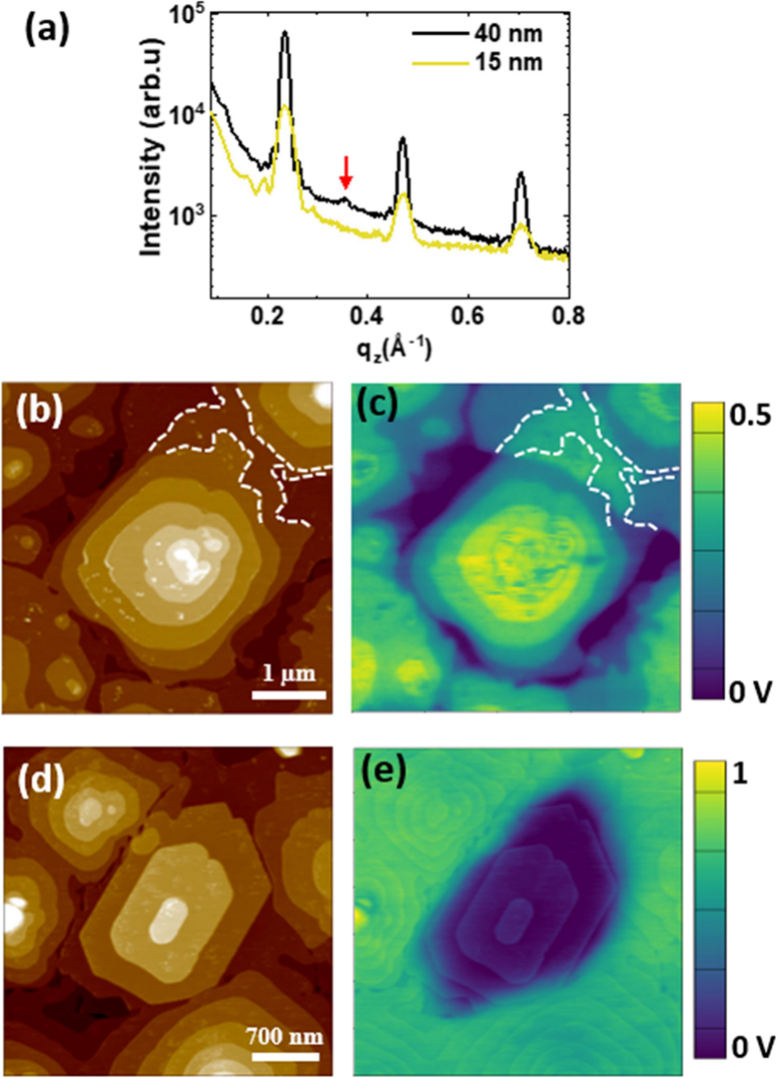
(a) Specular XRD spectra obtained for 15 and 40 nm-thick films. Topographic images of Ph-BTBT-10 films of the nominal thickness of 15 nm (b) and 40 nm (d). The SP maps simultaneously collected are displayed in (c) and (e), respectively. Some step profiles have been marked by dashed white lines in (b) and (c) to highlight a lack of correlation with the local SP in this region (see text).

The specular XRD spectra obtained for 15 and 40 nm-thick films (Figure 5a) show well-defined Bragg peaks at *q*_*z*_ = 0.235 Å^–1^ corresponding to a spacing of 2.675 nm, confirming a layered packing with one single-layer periodicity. GIWAXS data confirm the characteristic pattern of the HB BTBT packing (Figure S2). The small-intensity peak at *q*_*z*_ = 0.354 Å^–1^ (signaled by a red arrow) corresponds to the (003) Bragg peak of the bilayer lamellar structure (*d*_BL_ = 5.32 nm), confirming the formation of a small fraction of the crystalline BL bulk structure. Representative topographic data of Ph-BTBT-10 films of the nominal thickness of 15 and 40 nm are displayed in Figure 5b,d, respectively, along with the corresponding SP maps (c and e). In both cases, terraced mounds are observed, most of them corresponding to SL stacks in agreement with the XRD data. Interestingly though, the mound at the center of Figure 5d exhibits terraces with straight edges all separated by steps with a height of ≈5 nm (two molecular layers) and is therefore associated with the growth of a BL-crystallite. The presence of laterally segregated BL crystallites in the film is not rare (Figures S3 and S4), being responsible of the small fraction of crystalline BL observed by XRD (red arrow in Figure 5a). At this point, it is worth highlighting some interesting observations. As seen by the color scales in the KPFM maps, while SP increases with the number of SL in all visible mounds for the 15 nm-thick film (Figure 5c), for the 40 nm-thick film, SP is constant regardless of the number of SLs in the various mounds (lighter region in Figure 5e). We also note that the SP map in Figure 5c includes domains with different SP at the same terrace level (delimited by white dashed lines in Figure 5b,c). This fact arises from domain boundaries in buried layers that were formed during early growth stages, which might be due to SLs formed by domains of opposite polarity, as described before. Further examples of areas where the SP is uncorrelated with the morphology are given in the Figure S4.

### Modeling and DFT Calculations

**Figure 6:**
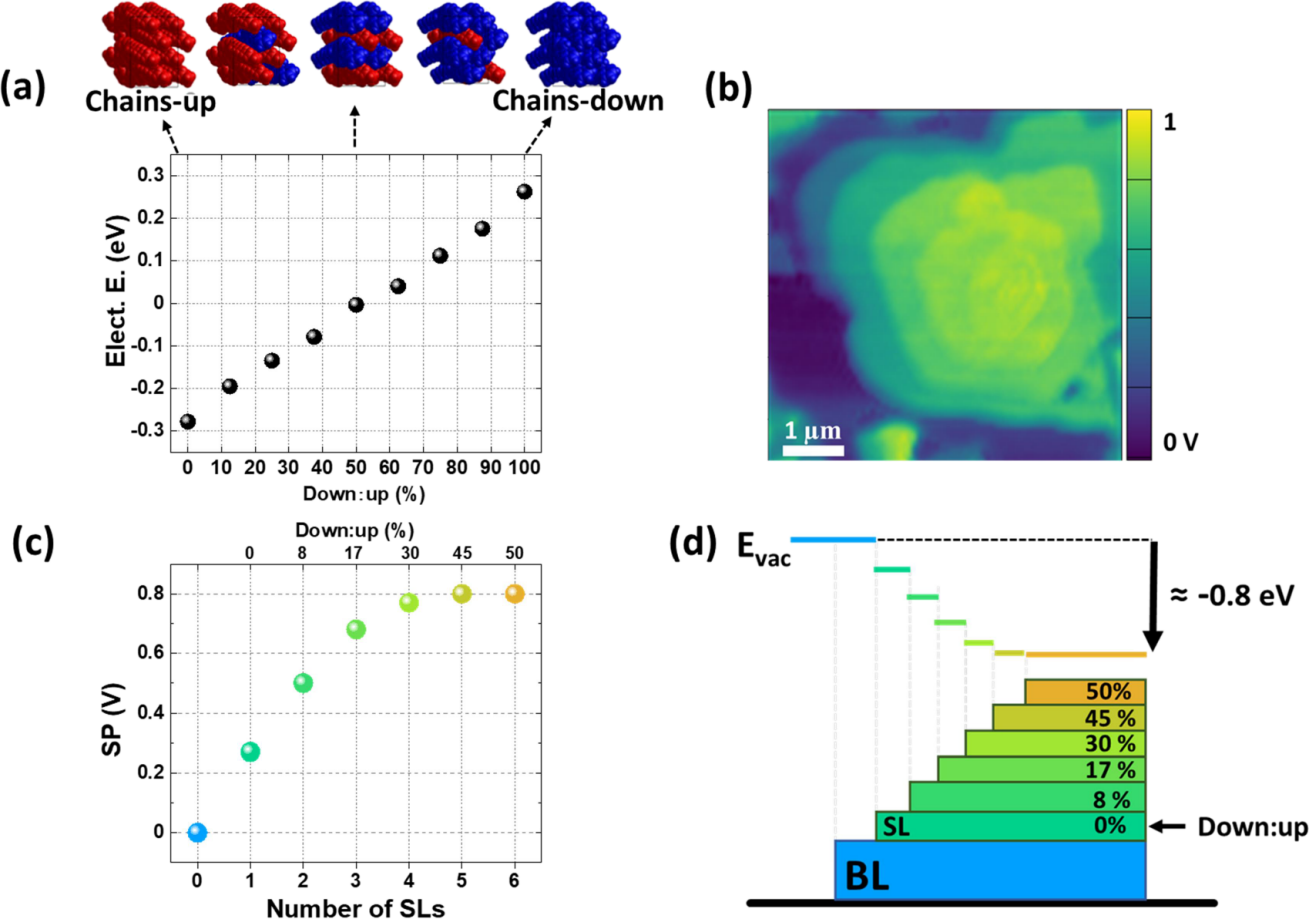
(a) DFT electron structure calculations: Electrostatic energy (vacuum level shift) calculated for a single monolayer as a function of the ratio between the two possible molecular orientations within the SL, i.e., chain-up (up) and chain-down (down). (b) KPFM map of a typical mound of stacked SLs. (c) Left axis: Experimental SP obtained from (b) as a function of the number of SL on top of the bottom bilayer, which is taken as the reference (SP_BL_ = 0). Top axis: percentage of down:up molecules for the diverse SLs estimated from (a) and the experimental difference in the SP by each added layer. (d) Illustrative cartoon indicating the percentage of down:up molecules in each layer and the effect on the vacuum level. The same color scale is used in all panels.

We use periodic DFT electronic structure calculations (see details in Experiments and Methods section) to model changes in the electrostatic potential of a perfect polar molecular layer, i.e., unidirectional orientation of the molecules, and in the presence of molecular disorder implemented by flipped molecules. The polar models were built from the smectic phase reported by Hofer et al. (Figure S5a),^21^ considering two possible dipole orientations. From now on, “up” and “down” configurations refer to the orientation outward and inward, respectively, of the alkyl chains of the Ph-BTBT-10 with respect to the substrate. The lattice vectors of the unit cell were increased by two along the in-plane directions to generate a supercell with an area of (12.1 Å × 16.6 Å) containing 8 BTBT molecules (25.1 Å^2^ per molecule, i.e., a full density packing). On that basis, we have considered a single molecular layer with several ratios of down:up molecules to mimic molecular defects in the form of molecular flipping, between the two limiting cases (100% “up” and 100% “down”), see Figure 6a. The averaged electrostatic energy was computed perpendicular to the film, taking into account the potential difference between the top and bottom film planes. The change in electrostatic energy corresponds to the shift in the vacuum level (i.e., work function change), which corresponds to an opposite shift in the SP observed by KPFM.

The calculations in Figure 6a show a linear evolution of the vacuum level shift when continuously changing randomly the relative ratio of the up and down configurations. For single monolayers in a pure-down configuration (down:up 100%), the change in the electrostatic energy is +0.264 eV, whereas switching the orientation of all molecular dipoles to a pure-up configuration (down:up 0%) results in a downshift of the vacuum level by −0.273 eV. Thus, the similar but opposite increase/decrease in the SP shift observed in some regions (Figure 3a–c) is explained by the formation of domains, each one made up of molecules adopting one of the two possible polar orientations. The dipole cancellation is almost perfect for a down:up ratio 1:1 (electrostatic energy ≈ −0.004 eV), corresponding to an antiparallel (mixed) configuration. As shown in Figure S5c, a polar single layer on top of a 1:1 mixed monolayer gives a similar shift as a polar free-standing single layer, thus implying a negligible role of depolarization. Similarly, no significant depolarization effect exists for a stacking of multiple layers, with the dipoles either adding up or canceling each other depending on their relative orientations (see Figure S5c). In particular, when all superimposed layers have the molecular dipoles aligned in the same direction, the total energy shift is virtually a multiple of the shift obtained for a single dipolar layer. Experimentally, this was not observed. The change in SP for a typical mound of packed SLs (Figure 6b) has been quantified and plotted in Figure 6c. The SP values measured at each terrace are depicted as a function of the number of SLs on top of the BL that is taken as a reference (SP_BL_ = 0). We first note that the SP value measured for the first SL on top of the BL is fully consistent with the expected downshift of the vacuum level from a pure-up configuration (down:up 0%). Remarkably, the experimental SP increases with the number of piled SLs until it saturates for ≈5–6 layers. Taking into account the thickness of the underlying bilayer at the substrate interface, the nominal thickness of the film with saturated SP is approximately ≈20 nm.

The measured nonlinear increase and saturation of SP is explained by a progressive increase in the amount of flipped molecules as the layer number increases. From Figure 6a, we estimate the percentage of down:up molecules that corresponds to the measured SP shift for each added SL (see also Figure S6). These estimations are given in the top axis of Figure 6c and the percentage of flipped molecules is indicated in the illustrative cartoon of Figure 6d. In such a scenario, from the parallel orientation (down:up 0%) in the first layer, a rising number of down-chain molecules is formed within each subsequent layer until, eventually, half of the molecules are arranged in an antiparallel fashion forming a mixed nonpolar SL (top center, Figure 6a). Hence, the thin-film phase reported for 60 nm-thick films (Figure 1b) is the nonpolar structure achieved, as the films thickens. Although the plot in Figure 6c has been measured in one particular mound, it represents the general behavior. Nevertheless, the SP saturation value may differ for different regions (between 0.8 and 1.1 V) due to differences in local crystalline quality (as demonstrated in Figures 3–5).

It is worth noting that from an energetic point of view, the unidirectional alignment of dipoles is unfavorable by +0.35–0.40 eV compared to the nonpolar antiparallel configuration (more stable). As we discussed later, its formation during growth can be considered a kinetically trapped surface-induced structure.

### Electronic Effects: Molecular Orbitals, Ionization Potential, and Vacuum Level

As we will see next, the local electrostatic potential data are key for a comprehensive understanding of the electronic density of states, as measured by UPS and the influence of thickness on the electronic properties of Ph-BTBT-10 films.

**Figure 7:**
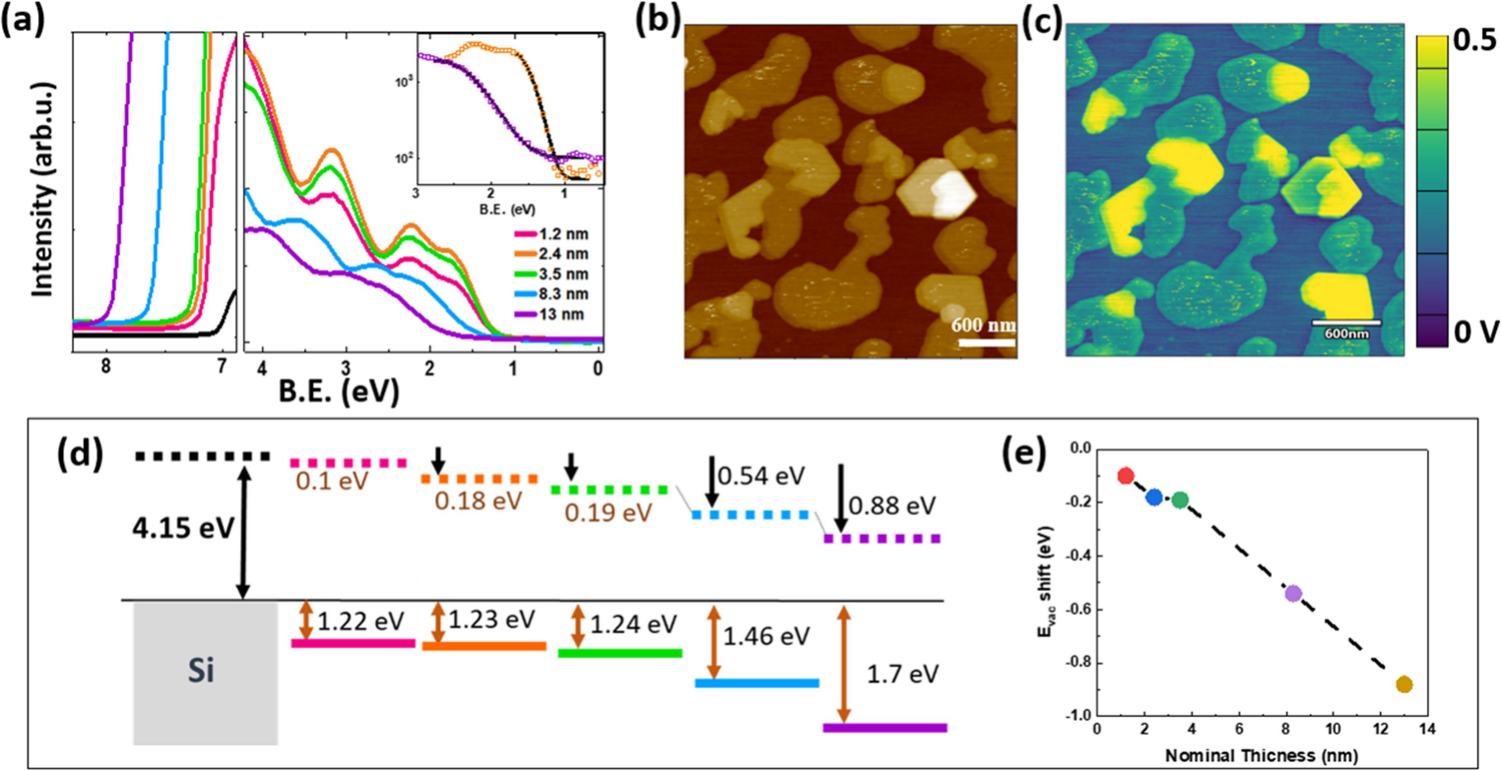
(a) In situ UPS measured for different amounts of Ph-BTBT-10 (nominal thickness indicated in nm) deposited at RT; SECO and valence band regions are depicted. (b) Topography and (c) surface potential map for a Ph-BTBT-10 nominal thickness of *x* nm deposited at RT. Under these conditions, islands of an incomplete BL leave uncovered substrate areas, while SL terraces form on top of the BL islands. Schematic illustration of the energy levels (d) and plot of the vacuum level shift (e) as a function of thickness, extracted from data in (a).

Figure 7 shows the UPS spectra obtained for different stages of the growth of Ph-BTBT-10 at RT. In particular, the corresponding secondary electron cutoff (SECO) and the highest occupied molecular orbital (HOMO) regions of the valence band (VB) are shown in Figure 7a. It is worth noting that during Ph-BTBT-10 growth at RT, nucleation of single molecular layers takes place on pre-existing BL islands before its completion (Figure 7b). Therefore, at the early stages of growth, the surface dipole of these nucleated SL terraces contributes to the surface-averaged SECO that results in a larger value than expected for a complete nonpolar BL. Up to a nominal thickness of 3.5 nm, there is no measurable alteration in the HOMO position and the SECO points to a condition of vacuum level alignment. Indeed, given that the Fermi level of the substrate is located within the gap of the OSC, charge transfer is not expected. As the thickness increases, a shift in the vacuum level (*E*_vac_) is accompanied by a shift to the higher binding energy of the HOMO and a visible broadening. For a deposition of 13 nm, a considerable decrease of the *E*_vac_ by 0.9 eV is obtained. Note that the saturation of the *E*_vac_, which is expected for a thickness of ≈20 nm, has not yet been reached; the 13 nm-thick film here can be considered a defective polar film. Figure 7d summarizes the evolution of both HOMO and *E*_vac_ with thickness. From the energy difference between HOMO and the SECO onset, a remarkable decrease in IP is estimated, from 5.27 to 4.97 eV for the total range of thickness studied. Although the HOMO onset region is well fitted with a Gaussian curve, indicating that there are no appreciable gap states, a clear broadening is observed. This is illustrated by the magnification of the frontier orbital region (inset Figure 7a) for nominal thickness of 2.4 and 13 nm, in which the corresponding fits (solid black lines) indicate a considerable broadening, with a change of the full width half-maximum (fwhm) of the Gaussian from 0.47 to 1.04 eV.

## Discussion

An outstanding observation previously reported^21^ and confirmed by this work is the formation of one bilayer (structural out-of-plane unit of the bulk phase) of Ph-BTBT-10 at the interface with the substrate. The almost negligible contribution to SP of the BL with respect to the substrate agrees with the zero net electrical dipole of this structure, where the opposite dipoles of each half-bilayer are canceled out. However, to the best of our knowledge, a demonstration that the lamellar stacking of individual molecular layers on top of this BL can be different from the established thin-film structure has not been reported so far. We have shown here the appearance of polar single layers akin to one-half-bilayer. Outstandingly, the unidirectional packing of molecules maximizes the interchain van der Waals interactions and enhances herewith the packing of the BTBT cores^26^ but, in terms of dipolar interactions, results in a considerable electrostatic energy cost. We hypothesize that the formation of the bottom BL templates the nucleation and growth of molecules with half-bilayer (unidirectional) packing. From this point of view, the reported polar polymorph is a surface-induced metastable structure that will be transformed into the bilayer crystalline structure (thermodynamically stable) upon thermal annealing. Although the alkyl-up configuration is favored, domains with the opposite orientation are also found. DFT calculations of the electrostatic potential yield a downshift of the *E*_vac_ by −0.273 eV (i.e., a positive shift of the SP) for a defect-free dipolar layer with chain-up orientation, which is in excellent agreement with the experimental value measured by KPFM for the first SL on top of the BL. The calculations also indicate that depolarization effects are negligible, so that a stacking of dipolar planes causes a net dipole moment perpendicular to the surface equal to the sum of each plane. Experimentally, the local SP shift measured as a function of the increasing number of stacked single layers does not escalate linearly but reaches a saturation value close to 1.0 V upon 5–6 layers. The observed changes in the SP can be adequately explained by the increasing proportion of flipped molecules within the stacked layers. As the film thickens, the assembly of alternating chain-up and chain-down molecules plus structural relaxation eventually leads to the nonpolar (1:1) thin-film structure.

**Figure 8:**
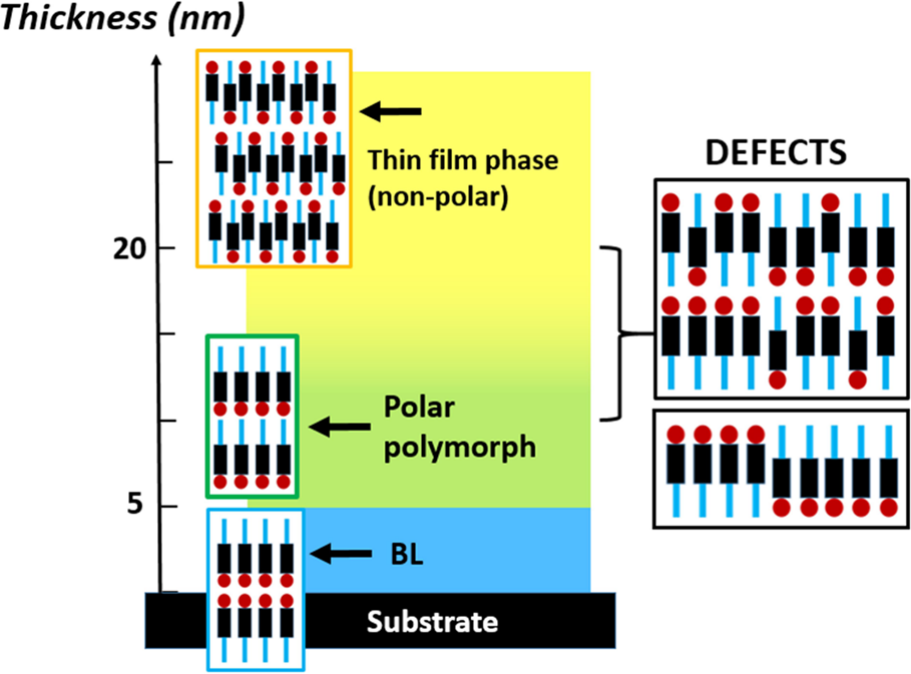
Cartoon illustrating the structural arrangement of Ph-BTBT-10 molecules during growth on a silicon substrate as a function of film thickness.

The overall scenario is illustrated in Figure 8. The structural evolution with thickness can be rationalized by simple electrostatic arguments. It is known that whenever there is a dipole moment in the repeating structural unit perpendicular to the surface, the surface energy diverges, meaning that the polar surface becomes unstable with crystal thickness along the polar crystallographic direction. This is the so-called “polar catastrophe”.^42,43^ For ionic crystals, there are several “polarity compensation mechanisms” to overcome undesired instability. In our case, inclusion of flipped molecules in the packing during the growth can be seen as a mechanism for electrostatic compensation, avoiding the “polar catastrophe” as the number of layers stacked increases.

Comprehending the impact that such thickness-dependent polymorphism has on the HOMO density of states in thin films holds significant importance due to its profound influence on gap states and energy alignment and, herewith, with important implications for the performance of devices. Indeed, the UPS study as a function of thickness reveals a rigid shift of the energy levels and hence a decrease in the overall work function, confirming the KPFM results and the effect of the net electrical dipole. Localized defects in the form of flipped molecules or grain boundaries amid opposing polar domains introduce energy differences among molecular sites, i.e., static disorder. Although the impact that extrinsic static disorder has in the electronic properties of organic semiconductors (by producing gap states) has been demonstrated,^44−46^ here we provide direct evidence of the dramatic consequences of structural imperfections related to the polar character of the asymmetric BTBT molecule. Hence, for Ph-BTBT-10 thin films, the predominant source of energetic disorder is electrostatic in nature, stemming from the inherent electrical dipole of the molecule and causing a considerable broadening of the HOMO (here from 0.47 to 1.04 eV) as the film thickens. KPFM has been demonstrated to be an essential tool to identify the nature and spatial distribution of electrostatic defects in the film. In addition to structural defects in the form of individual flipped molecules (point defects), the formation of sizable domains, i.e., separated assemblies of one or the other possible polar orientations leads to heterogeneities in the electrostatic potential either in the form of islands (Figure 3c) or in small lateral scales (Figure 3d). Interestingly, in the case of buried (intercalated) domain boundaries, intriguing SP maps that appear uncorrelated to the morphology can be found (Figures 4 and 5). Furthermore, from combined KPFM and XRD data, we have shown that even when the film shows a predominant SL-layered structure, a small fraction of crystallites with the BL structure may also appear laterally segregated and remain embedded within the film (Figures 4, 5, and S4), causing a large spatial variation in the work function.

In this context, it is worth mentioning that Resel and co-workers have proposed a specific type of structural disorder in which the head-to-head stacking within the crystalline bilayer includes a fraction of flipped molecules. In view of our results, we believe that such defective BL packing will also lead to electrostatic disorder.^20^

The observed changes in IP are not expected from a purely electrostatic picture, according to which the HOMO should closely follow the vacuum level resulting in a constant IP; however, any speculation is avoided here since a reliable interpretation of UPS can be particularly difficult for energetically inhomogeneous surfaces at the nanoscale.^48^

Overall the findings of this work are projected to exert a significant influence on device performance, unraveling factors affecting the field-effect mobility, the threshold voltage, and contact resistance, which certainly warrant further investigation. Given that the formation of polar structures and different kinds of defects are expected to be very sensitive to the processing protocol, the characterization of polymorphism in thin films should go beyond a simple specular XRD and morphological analysis. X-ray characterization lacks the necessary spatial resolution and does not allow us to probe easily the coexistence of the different polymorphs at microscopic scale.

The stacking of single molecular layers on top of the bottom bilayer is the result of a subtle interplay of electrostatic and van der Waals interactions, which are expected to be also in play for other asymmetric BTBT derivatives explored for their high potential in high mobility OFETs.^15,30^

## Conclusions

In this study, we unveil the great level of structural intricacy of Ph-BTBT-10 thin films that goes beyond the reported single-layer to bilayer transformation model. We demonstrate that between the double layer structure located directly at the substrate surface and the metastable thin-film phase, there is a structural phase that consists of the packing of polar single layers. DFT electronic structure calculations quantify a shift in the vacuum level by −0.273 eV per layer, assuming a defect-free polar layer of oriented chain-up molecules, which is in excellent agreement with the measured SP. Experimentally, the shift is reduced for subsequent layers and is explained by an increase in the percentage of flipped molecules. The net dipole cancellation (nonpolar) is almost exact for the alternate (1:1) ratio, reaching above a critical film thickness of ≈20 nm. We conclude that in polar organic semiconductor thin films, flipping of molecules is the polarity compensation mechanism.

Our study underscores that KPFM is a valuable tool to evaluate electrostatic disorder and the conceivable emergence of polar polymorphs in thin films. We identify a variable number of structural defects in the form of individual flipped molecules (point defects) or sizable polar domains (with chain-up and chain-down configurations). The actual film structure has dramatic consequences in the electronic properties, in particular, a decrease in the work function of the film with increasing thickness (up to ∼1 eV) and a broadening of the HOMO attributed to electrostatic disorder.

The synthesis of asymmetric compounds by molecular engineering is a recently developed strategy for obtaining layered crystalline OSCs with high field-effect mobility. The findings of this study imply that the development of polar assemblies may represent a common issue for other asymmetric BTBT derivatives and deserve consideration for understanding the structure–property relationships in OFETs. A precise determination of the possible formation of polar assemblies close to the interface is pivotal for the strategic design and advancement of high-performance OFETs.

## Experiments and Methods

### Molecular Film Growth

Ph-BTBT-10 (purchased from TCI Chemicals) films were grown from the vapor phase with a deposition rate of ≈2 Å/min onto the native oxide surface of Si(100) substrates (p-type) under high vacuum conditions (10^–7^ mbar) and at different substrate temperatures, ranging from 25 to 90 °C. The substrates were cleaned by sonication in acetone and ethanol for 10 min each, followed by 10 min of UV-ozone cleaning. The growth rates were monitored by a quartz crystal microbalance (QCM). The amount of deposited molecules is given as the nominal thickness in nm.

### AFM and KPFM

Topographic and local SP measurements were performed at RT under a N_2_ gas atmosphere to minimize moisture effects, using a Cypher ES Environmental AFM instrument, from Asylum Research (Oxford Instruments). KPFM measurements were conducted in amplitude modulation (AM-KPFM) with an AC voltage of 1 V at the frequency of the first eigenmode in a two-pass procedure (Nap mode). During the first pass, a topographic contour line is recorded in dynamic mode (constant amplitude). In a second pass, the KPFM is obtained while the mechanical excitation is switched off and the tip is driven to follow the topographic contour but approached toward the sample surface by a selected height difference. For the setup employed, where the voltage bias is applied to the tip, higher (lower) SP corresponds to lower (higher) surface work function. The shift in E_vac_ in units of eV corresponds to the absolute value with the shift of SP in units of volts but has an opposite sign. Silicon tips with a Ti/Ir (5/20 nm) coating and a nominal spring constant of 2.8 (N/m) were employed.

### UPS

VB and secondary electrons cutoff (SECO) spectra were measured by ultraviolet photoelectron spectroscopy (UPS) using an ultrahigh-vacuum (UHV) system equipped with a SPECS Phoibos 150 hemispherical energy analyzer and a Helium lamp (*h*ν = 21.22 eV). To have access to the work function from the SECO, the corresponding spectra were taken with the samples biased at −10 V. A Au(111) single crystal was used as a reference for Fermi energy determination. The full width at half-maximum of the HOMO has been used as an indicator of disorder, which was extracted by using the Gaussian distribution to fit the first HOMO from each spectrum.

### XRD

XRD in specular geometry was performed with a D8 DISCOVER multipurpose X-ray diffractometer (Bruker) using a wavelength of 1.542 Å (Cu Kα). The 2D diffraction pattern was acquired by GIWAXS at the BL11-NCD-SWEET beamline of the ALBA Synchrotron (Spain) using a photon energy of 12.4 keV with an incident angle of 0.13°.

### Theoretical Calculations

The theoretical investigation of the electronic properties (electrostatic potential and energy levels) was done at the DFT level using the SIESTA code.^49,50^ The calculations were carried on using the PBE functional with a real-space grid cutoff of 350 Ry, a DZP (double-ζ + polarization) orbital basis set, and a k-point grid of (3 × 2 × 1). The organization of the BTBT films was modeled based on the crystalline unit cell obtained by Hofer et al. using MD simulations.^19^ From the bulk structure, we have generated several conformations to model SL structures with several “down:up” ratios. The electrostatic potential was computed from the converged electron density using the Macroave tools.^47^ A large vacuum region was considered perpendicular to the layer surface, and a dipolar correction was applied to cancel the cell self-interaction.

## Supporting Information Available

Additional topographical and surface potential maps obtained by KPFM illustrating the coexistence of different polymorphs, GIWAXS data as well as further details of the calculations (PDF)
