# Supplementary material for: Polar Polymorphism: A New Intermediate Structure toward the Thin-Film Phase in Asymmetric Benzothieno[3,2-b][1]-benzothiophene Derivatives
Source: Chem Mater. 2023 Dec 26;36(1):585–95. doi: 10.1021/acs.chemmater.3c02926 (PMC10783425; doi:10.1021/acs.chemmater.3c02926)
Supplement: Supplementary file 1 — cm3c02926_si_001.pdf [file cm3c02926_si_001.pdf]

Supporting Information

Polar polymorphism: a new intermediate structure towards the thin-film phase in asymmetric benzothieno[3,2-b][1]-benzothiophene derivatives

Polar polymorphism: a new intermediate structure towards the thin-film phase in asymmetric benzothieno[3,2-b][1]-benzothiophene (BTBT) derivatives

Shunya Yan,[1] David Cornil, [2] Jérôme Cornil, [2] David Beljonne,[2] Rogger Palacios-Rivera,[1] Carmen Ocal[1] and Esther Barrena[1]

[1] Instituto de Ciencia de Materiales de Barcelona (ICMAB-CSIC), Campus UAB, Bellaterra, E-08193 Barcelona, Spain.
[2] Laboratory for Chemistry of Novel Materials, University of Mons (UMONS), 20 Place du Parc, 7000 Mons, Belgium

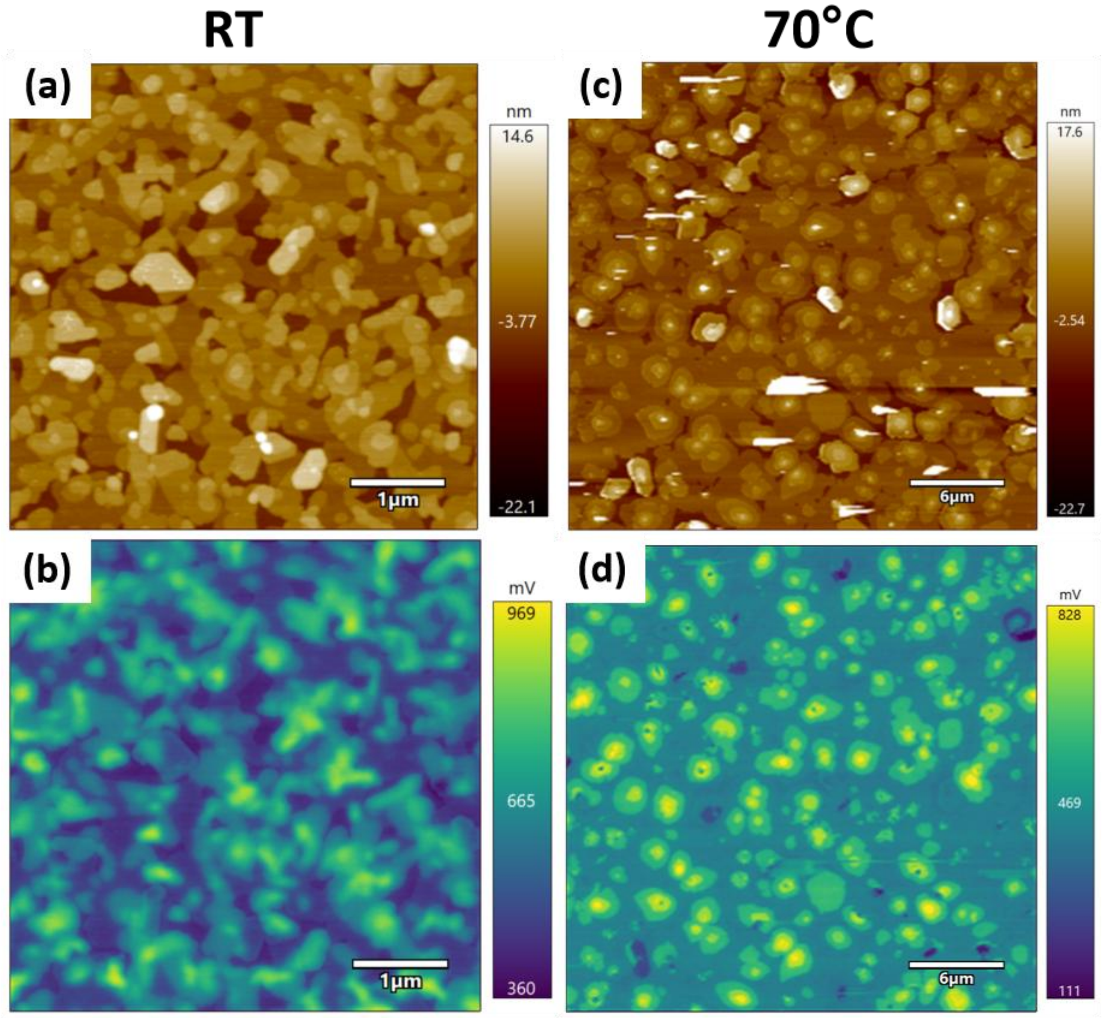

Figure S1. (a) Topographic image (5 µm x 5 µm) and (b) corresponding surface potential map for Ph-BTBT-10 deposited at room temperature. (c) Topographic image (30 µm x 30 µm) and (d) surface potential map for Ph-BTBT-10 deposited at a substrate temperature of 70 °C. The substrate is the Si(100) with native oxide. A higher substrate temperature favors the formation of larger terraces during growth; morphologies with larger terraces allow a better quantification of local differences in the surface potential. Nominal thickness is ≈6-7 nm in both cases.

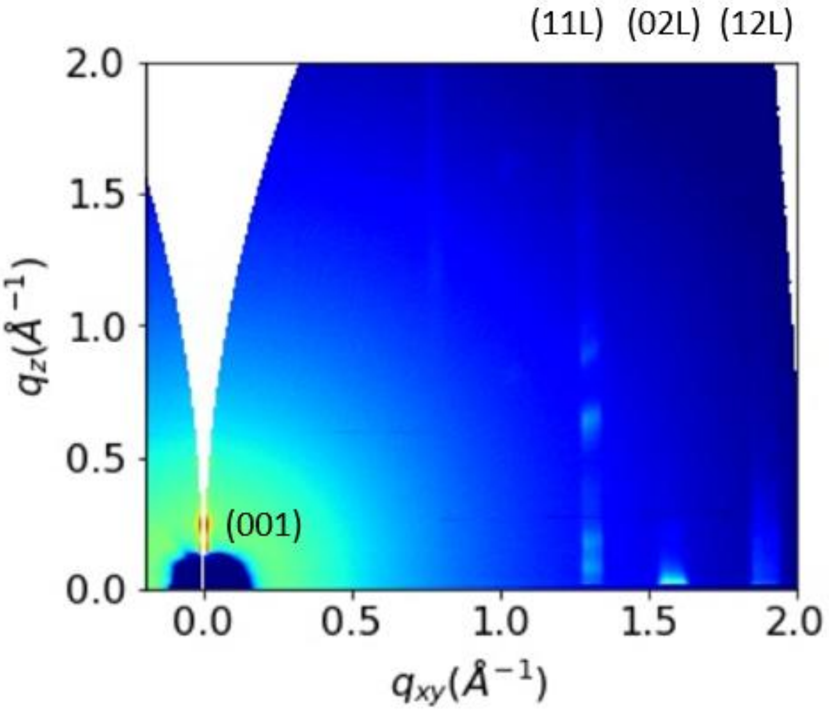

Figure S2. GIWAXS data measured for Ph-BTBT-10 (thickness≈ 15 nm) on SiO_2 showing the characteristic diffraction pattern of the herringbone BTBT packing.

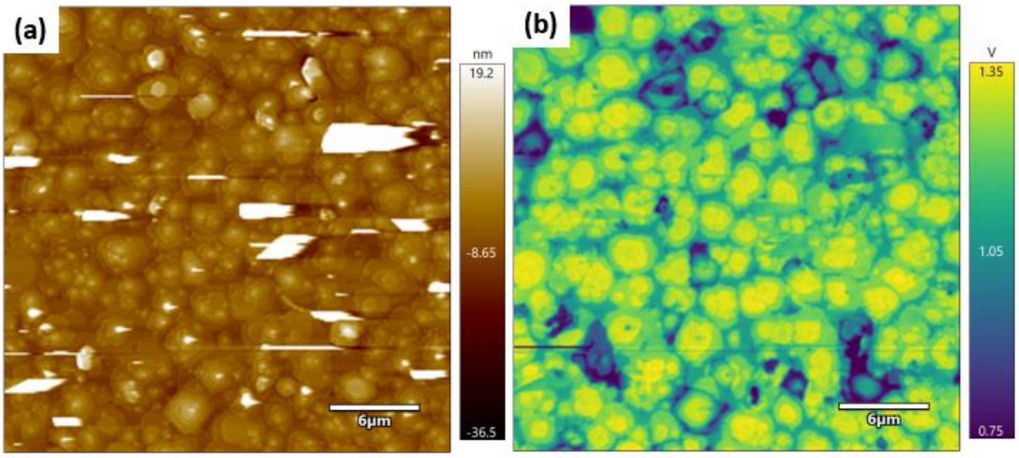

Figure S3. (a) Topographic image and (b) corresponding surface potential map of Ph-BTBT-10 (nominal thickness of ≈ 15 nm) grown on SiO_2 with the substrate kept at 70°C. As described in the text, most of the terraced mounds in the film correspond to stacks of SLs. There are, however, some mounds formed by BL-stacking, which can be distinguished by a lower SP.

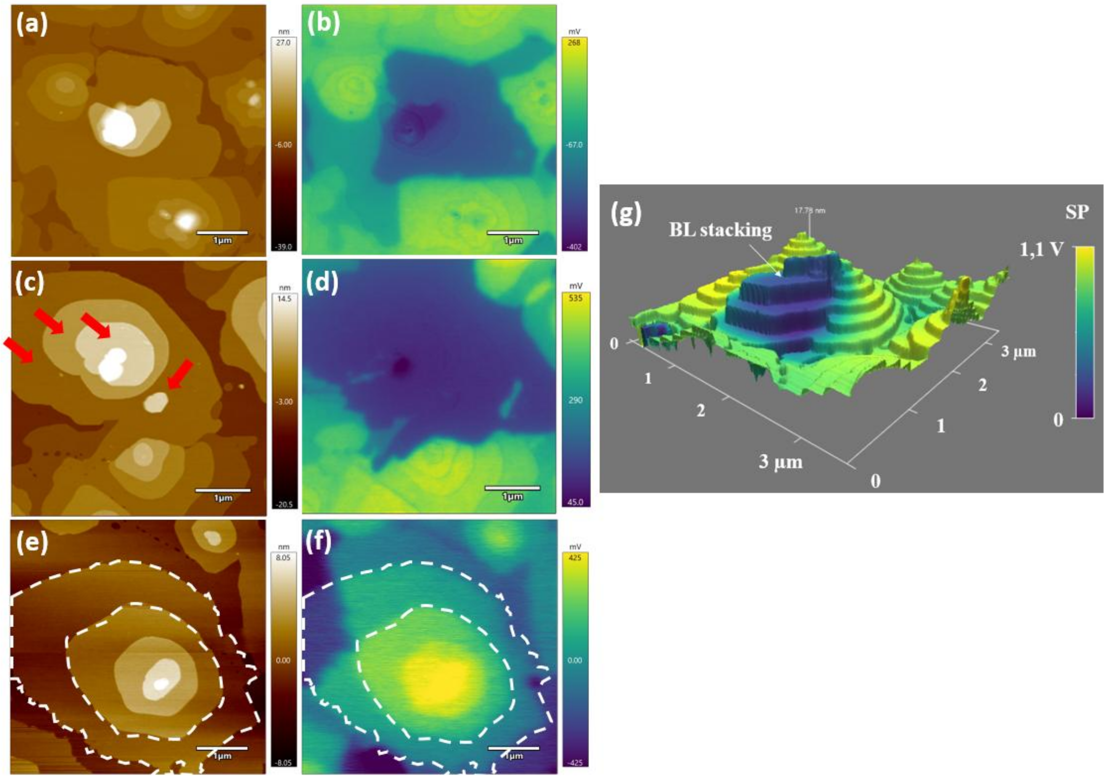

Figure S4. Topographic images (a, c, e) and the corresponding surface potential maps (b, d, f) of Ph-BTBT-10, with a nominal thickness of 12-16 nm, deposited with the substrate held at ≈ 70 °C. It can be observed that the surface potential maps are not correlated with all morphological features. In some cases, as in e and f, the same terrace (delimited by dashed lines) exhibits more than one value of surface potential due to buried single layers with domain boundaries with opposite (up and down) dipolar orientations. In other regions, as in c and d, terraces with same surface potential are associated to a region of non-polar BL structure (i.e., terraces with a double step height are indicated by red arrows). (g) Three-dimensional visualization of the merged topographic and SP data for a 40 nm-thick film. The main mound is formed, in fact, by two coalescent parts with BL and single layer structures, which are clearly distinguished by double (left mound part) and single (right mound part) step heights, respectively, and by the large difference between the SP for each of the stacking sequences.

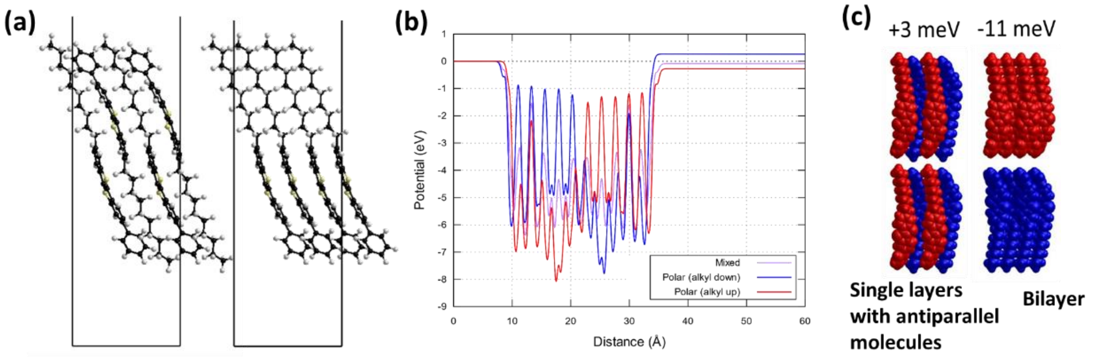

Figure S5. (a) Unit cell used for the calculations discussed in the manuscript. The molecular models are built from the smectic phase reported by Hofer et al. [ref. 17]. (b) Variation of the potential for full mixed vs polar (alkyl up and alkyl down) as a function of the distance to the substrate surface. Up and down configurations refer to the orientation outwards and inwards, respectively, of the alkyl chains of the Ph-BTBT-10 with respect to the substrate. (c) Left: Side view of a stack of two identical layers with a (1:1) ratio of up and down molecular conformations (50%). Right: Side view of a bilayer (BL) made of a pure chain-up layer on top of a pure down layer. The indicated values correspond to the potential shifts in these cases. Colors in (b) are consistent with the used in (c).

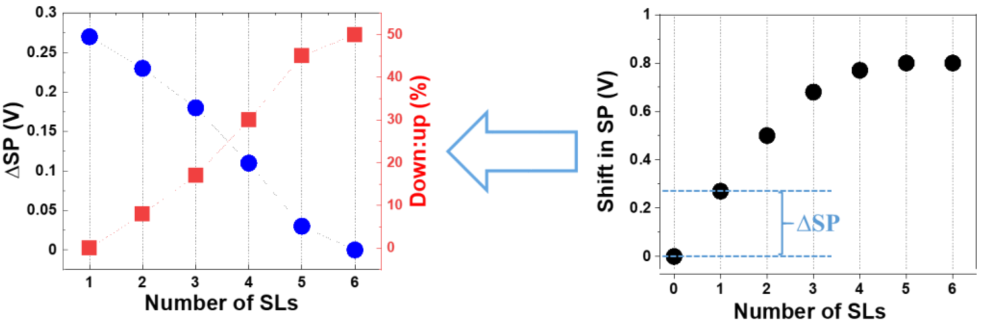

Figure S6. The surface potential increase (ΔSP) arising from the addition of each SL of Ph-BTBT-10 is estimated from the evolution of the SP vs. number of stacked SLs (right) shown in Figure 6c of the manuscript. The ΔSP values thus obtained (blue symbols) are compared with those expected from the calculations in Figure 6a for one molecular layer with a given percentage of down:up molecules. This procedure allows estimating the down:up percentage of each SL (red symbols). The percentage indicates the ratio of flipped molecules (defects), i.e., 0% corresponds to a perfect polar layer of up-chain molecules.
